# Case Report: Xenogeneic mesenchymal stem cell secretome for the treatment of feline chronic gingivostomatitis

**DOI:** 10.3389/fvets.2025.1603383

**Published:** 2025-06-19

**Authors:** Angel Almendros, Omid Nekouei, Claudia Moores, Robert Jesky, Kerstin Baiker, May Tse

**Affiliations:** ^1^Department of Veterinary Clinical Sciences, Jockey Club College of Veterinary Medicine, City University of Hong Kong, Kowloon, Hong Kong SAR, China; ^2^Department of Infectious Diseases and Public Health, Jockey Club College of Veterinary Medicine, City University of Hong Kong, Kowloon, Hong Kong SAR, China; ^3^Veterinary Medical Centre, City University of Hong Kong, Kowloon, Hong Kong SAR, China; ^4^Hong Kong Regen Medtech Ltd., HKSTP, Hong Kong, Hong Kong SAR, China

**Keywords:** feline chronic gingivostomatitis, immunomodulation, mesenchymal stem cells, oral mucosa, secretome, supernatant, xenogeneic

## Abstract

This case series describes the treatment of eight cats with mesenchymal stem cell (MSC) secretome, a therapeutic modality never used before for refractory feline chronic gingivostomatitis (FCGS). The cats were free of systemic illness and were not on immunomodulators during treatment. All cats received two to three treatments of MSC secretome through intramucosal injections given 3 months apart. White cell count and globulins, stomatitis disease activity index (SDAI), and histopathology were used pre- and post-treatment to assess the response to treatment. Of the eight selected cases, resolution of clinical signs and significant improvement of lesions were reported in two cats. Six cats did not have a substantial clinical response, and lesions remained mostly unchanged. Six cats had a reduction in globulins, and five cats increased in weight, respectively, after treatment. SDAI decreased in all the cats treated. The two cats that had resolution of clinical signs had the most significant decrease in serum globulins and SDAI, and both increased in weight. No adverse effects or chemistry and hematological abnormalities directly associated with the use of MSC secretome were observed in any of the cats, suggesting human-derived MSC secretome can be safely used in cats. Increases in weight and reductions in SDAI and globulinaemia were observed in a subset of cats after treatment, suggesting an immunomodulatory effect and downregulation of proinflammatory factors. Well-designed clinical trials are recommended to verify the observed effects in this study and to evaluate long-lasting clinical benefits or potential side effects of the treatment.

## 1 Introduction

Feline chronic gingivostomatitis (FCGS) is an immune-mediated oral mucosal inflammatory disease that may affect up to 12% of the cat population in first opinion practice (1). FCGS has a multifactorial aetiology but was initially believed that cats suffering from this ailment displayed an atypical immune reaction to dental plaque, resulting in extensive inflammation of the oral mucosa leading to significant mucositis and often presenting ulcerative or ulcero-proliferative lesions caudally and lateral to the palatoglossal folds of the oral cavity (2, 3).

Due to its unclear cause, FCGS is a challenging condition to treat. It is suggested that an inappropriate immune response to an unknown antigenic stimulus occurs, with proposed causes including feline calicivirus infection, other viral and bacterial infections, and cohabitating multi-cat households (4, 5). Recent reports are assessing the association of microbiome and FCGS (6, 7). Current treatment options for FCGS often involves a radical partial or full mouth tooth extraction. In addition, supplemental therapy including immunosuppressors, immunomodulators, anti-inflammatories, analgesics, and antibiotics is frequently used as part of the standard treatment protocols, needed lifelong in refractory cases (5, 8–10).

There is abundant research on the potential benefits of immunomodulation using mesenchymal stem cell (MSC) therapy for FCGS (11–15). Stem cells are undifferentiated cells that secrete growth factors and cytokines and that differentiate into cells with specialized functions (16, 17). Allogenous or autologous stem cells hold promise for improving the management of feline chronic gingivostomatitis when used after extractions (11–15). Other than its use for FCGS, stem cell therapy products have been used safely in dogs and cats in other conditions where immune modulation is involved including renal, pulmonary, intestinal, and neuromuscular disease (18–23). The use of xenogeneic stem cells or secretome in companion animals, however, has been reported in fewer cases (24, 25).

A large part of the therapeutic effectiveness of MSCs comes from the release of paracrine factors (16, 26–28), which are also present in the secretome, also referred to as supernatant, and include soluble factors such as cytokines, chemokines, and growth factors and non-soluble factors such as extracellular vesicles (EVs) and exosomes (16, 27–29). These have a functional effect on monocytes, dendritic cells, T cells, B cells, and natural killer cells with immunomodulatory effects through TGF beta, indolamine-2,3 dioxygenase (IDO), PGE2, IL-10, and TNF-stimulated gene 6 (TSG-6) (30–34). Some of these benefits include angiogenesis, wound healing, migration, and homing, inhibiting proliferation and an anti-inflammatory effect (16).

Researchers and clinicians continue to explore effective treatment options that can alleviate the symptoms and improve the long-term prognosis of cats suffering from FCGS. Collective evidence supports the use of MSCs for FCGS in cats following radical dental extractions (35). Feline chronic gingivostomatitis is a complex disease that requires a comprehensive multi-modal treatment approach and could benefit from the inclusion of novel regenerative medicine therapies. Therefore, in this case series, we described the treatment details and outcomes of eight cats treated with secretome from human-derived MSCs for FCGS. To the best of our knowledge, this is the first time this therapeutic modality has been reported in cats.

## 2 Materials and methods

### 2.1 Study design

In this case series, we selected eight cats treated with a novel therapy in veterinary medicine for FCGS. The study protocol was approved by the Animal Ethics Committee of the City University of Hong Kong (reference A-0831) and the Department of Health of Hong Kong for animal studies [reference number (22–103 in DH/HT&A/8/2/5 Pt.9)]. All clients owning the cats participating in the study had been informed of potential benefits and side effects involved with treatment by the attending clinician and had signed a consent form.

### 2.2 Case selection

Eight cats had been treated for FCGS in the same manner at the Veterinary Medical Centre of the City University of Hong Kong (VMC) from October 2022 to May 2023. Cases were included in the study if they had suffered from chronic gingivostomatitis. All cats had undergone partial (premolar and molar teeth) or full mouth (including canines and incisors) extractions at least 6 months prior to treatment and had the respective radiographic evidence. The selected cats were not taking on or had stopped taking any other immunomodulator or anti-inflammatory drugs, such as cyclosporine or corticosteroids, at least 10 days before treatment with MSC secretome. A Stomatitis Disease Activity Index (SDAI) score had been used and was equal or greater than 15 (0–30) in all cats included in this report. This score had been calculated by assessing inflammation in different parts of the oral cavity as well as by owner’s evaluation of parameters such as discomfort, grooming, appetite, and activity (2). The cats were otherwise visibly healthy, and a blood test had been performed no longer than 3 months prior to the treatment or at the time of treatment, showing no other obvious systemic comorbidities.

### 2.3 Mesenchymal stem cell secretome

The MSC secretome had been obtained by the only supplier of such product available in Hong Kong in highly concentrated 100 mg, cell-free, lyophilized form ready for dilution and administration. The secretome was comprised of various bioactive factors secreted from the MSCs which were EGF, VEGF, HGF, FGF2, and KGF. Each vial contained 100 mg of protein, intended to be reconstituted in 1 mL of water for injection. The secretome was prepared from serum and xeno-free medium.

#### 2.3.1 Cell culture and expansion

Human adipose-derived mesenchymal stem cells (hu-AdMSCs) were cultured in 6-well CellBIND plates (Corning) until they reached confluency of ≥ 80%. The medium was carefully aspirated, and 2 mL of TrypLE™ Express Enzyme (1X) was added to detach the cells. Plates were incubated for 5 min at 37°C, with gentle shaking after 2 min. Detached cells were observed under a phase-contrast microscope, and the detachment process was halted by adding 2 mL of R: Stem Medium for hMSC High Growth (Rohto Advanced Research). The cell suspension was transferred to a 50 mL conical tube and centrifuged at 400 g for 5 min. The cell pellet was resuspended in 2 mL of pre-warmed R: Stem Medium and counted using a LUNA-II™ Automated Cell Counter. Cells were then plated at a density of 10,000 cells/cm^2^ in 75 cm^2^ Corning CellBIND flasks and expanded to passage P-02 and then transferred to two-layer cell factories for expansion before initiating conditioned medium collection.

#### 2.3.2 Cell viability analysis

Cell viability was assessed at each passage using trypan blue staining. Viable cells were counted using the LUNA-II™ Automated Cell Counter, and only cultures with viability greater than 80% were used for subsequent passaging.

#### 2.3.3 Conditioned medium preparation and collection

MSCs at P-03–P05 were seeded into two-layer cell factories at a density of 5,000 cells/cm^2^ and cultured to 85–95% confluency in R: Stem Medium for hMSC High Growth (Rohto Advanced Research) for 7 days in standard normoxic conditions (5% CO2, 37°C). Conditioned medium (CM) or secretome, was collected at two time points (3 and 7 days *in vitro*). CM was centrifuged at 4200 rpm for 10 min to remove cell debris, followed by filtration through 0.22 μm PES filters (Millipore Steritop®). The filtered CM was stored at −20°C for subsequent processing.

#### 2.3.4 Concentration of conditioned medium proteins

Filtered CM from passages 3–5 was pooled to create a homogeneous batch. The CM was concentrated up to 30-fold using 3 K MWCO ultrafiltration centrifugal devices (Pierce™ Protein Concentrator PES, 3 K MWCO, Thermo Scientific™). Centrifugation was performed at 4500 rpm at 4°C. The concentrated retentate was combined and prepared for lyophilization.

#### 2.3.5 Lyophilization of concentrated conditioned medium

The concentrated CM was placed in 50 mL Falcon tubes and flash-frozen in liquid nitrogen (LN2). Samples were lyophilized using an SP VirTis BenchTop Pro lyophilizer (SP Scientific) at a condenser temperature below −80°C for 48–72 h. The final lyophilized product was then stored at −20°C until further use.

### 2.4 Study design protocol

All the cases included in this study had visited VMC on three or four occasions. The visits had been approximately 1 month, 3 months, and 6 months after the initial visit following manufacturer’s suggestions. These visits were designated as visit 1 (V1), V2, V3, and/or V4, respectively.

On V1, cats had a physical exam and a blood test to assess a complete blood cell count (CBC) and a biochemistry blood panel profile. All cats had also had retroviral assessment via a serology test (SNAP FIV/FeLV Combo Test, IDEXX Laboratories Inc., Taipei, Taiwan) as this is common practice as a screening test, especially for cats with FCGS before surgical extractions.

**Figure 1:**
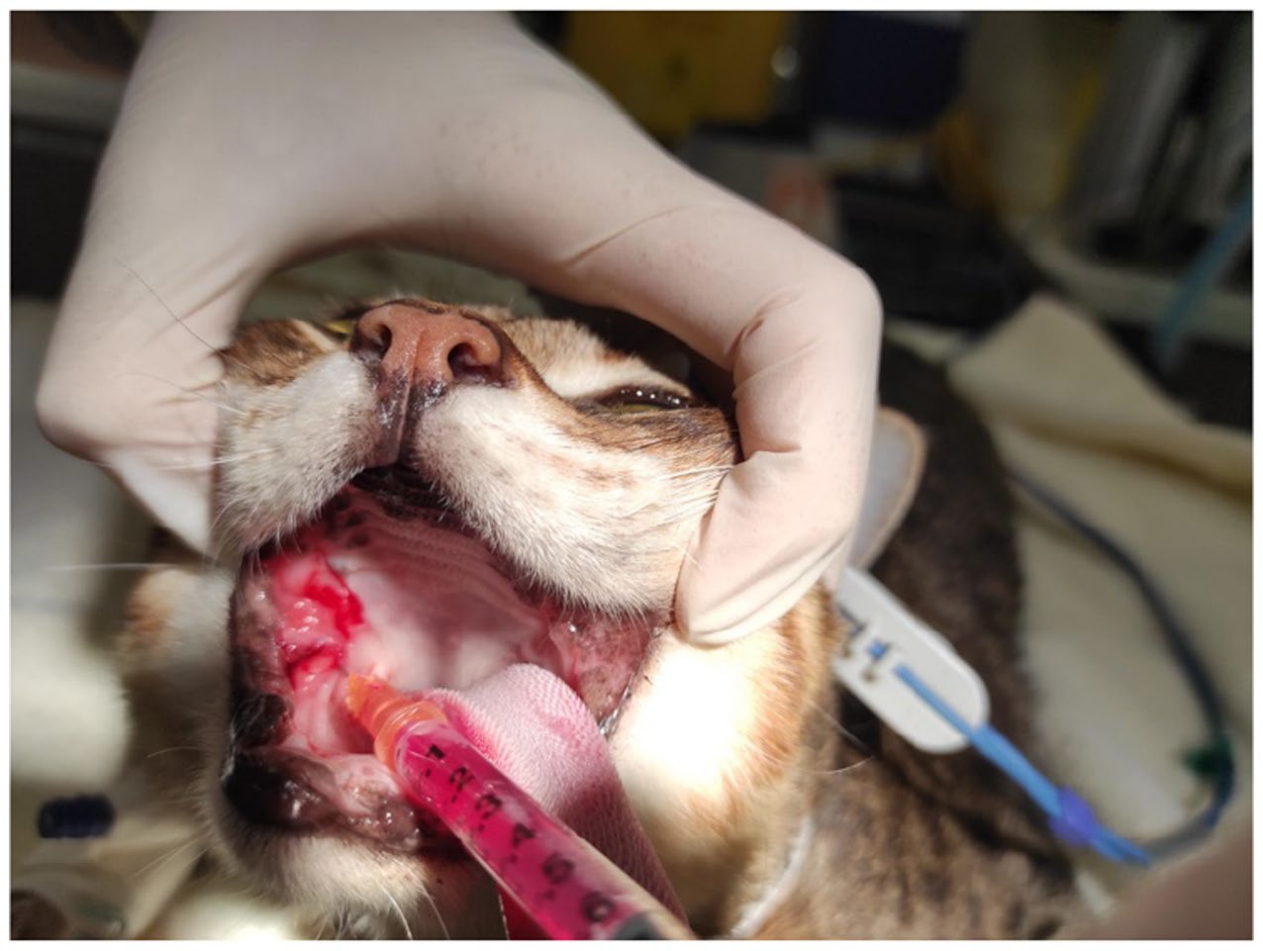
Intramucosal injection of MSC secretome in a cat suffering from FCGS.

Cats were sedated with medetomidine (40–80 μg/kg) [Domitor, Zoetis, Leatherhead, UK] and butorphanol (0.2–0.3 mg/kg) [Torbugesic, Zoetis, Leatherhead, UK] or with alfaxalone (3–5 mg/kg) [Zoetis, Leatherhead, UK] intravenously. Oral exam and a SDAI assessment were performed and recorded. As part of a parallel study, a small 2–4 mm biopsy sample was taken from the palatoglossal affected region in each cat using atraumatic tissue forceps and iris scissors. All cats were treated afterward with MSC secretome, crystalized form containing 100 g dissolved in 1 mL of water for injection and injected in equal parts in a 4-quadrant pattern on right dorsal, left dorsal, left ventral, and right ventral affected palatoglossal mucosa (Figure 1). After the procedure, the cats were reversed with intramuscular atipamezole hydrochloride (200 μg/kg) [Antisedan, Zoetis, Leatherhead, UK]. The procedure lasted approximately 20 min in total. No other medication other than oral (sublingual) buprenorphine (0.02 mg/kg q 8 h) [Temgesic, Schering-Plough, New South Wales, Australia] for analgesia purposes was given. The latter was prescribed for up to 5 days following the procedure for each cat. No antibiotics or other anti-inflammatory drugs were prescribed in between visits, but buprenorphine was prescribed occasionally if cats were reported to show oral discomfort.

The eight cats included in the study had a second visit (V2) approximately a month later that included a SDAI assessment and a CBC and a serum chemistry profile.

All eight cats had records of a third visit (V3) approximately 3 months after initial treatment that included another blood test panel followed by another MSC secretome injection. They had been sedated and had an oral exam and a SDAI assessment. A small 2–4 mm biopsy sample had been also taken. Following the biopsy sampling, cats were treated with MSC secretome using the same crystalized formulation, 100 mg diluted in 1 mL of water for injection, that was applied in the same 4-quadrant injected manner into the affected palatoglossal mucosa. Analgesia had been provided in the same manner as on V1.

Six cats had returned for a fourth visit (V4) and had a blood sampling, sedation, biopsy sampling, and post-procedure analgesia in the same manner as on V1 and V2, respectively.

#### 2.4.1 Histologic examination

Biopsy tissue samples were fixed in 10% neutral-buffered formalin at VMC and then submitted to VDL for further analysis. After fixation, representative sections were cut into blocks for routine processing, where 5-μm sections were counted, mounted, and stained with haematoxylin and eosin (H&E) and toluidine blue (TB) according to standard protocols. All stained histopathology slides were reviewed by two pathologists (KB and MT). Evaluation and qualitative grading of the inflammatory cell infiltrate was performed on H&E-stained slides. The predominant inflammatory cell types were reported (Table 1).

**Table 1:** Histopathological data from cats suffering from FCGS that were treated with MSC secretome.

Cat ID	Visit (V) number	Histopathology changes	Severity^	Exocytosis^	Erosion^	Ulceration^	Epithelial hyperplasia^	Stromal oedema^	Neutrophils#	Mott cells/hpf+	Mast cells/hpf+
1	1	LP	4	3	4	0	2	2	1	12	71
	2										
	3	LP	4	3	4	4	2	2	2	2	43
2	1	LP	4	2	4	4	0	2	2	50	5.7
	2										
	3	LP	4	3	4	2	3	2	3	9	4.2
3	1	LP	4	3	3	3	3	2	2	9	15.4
	2										
	3	LP	4	4	4	3	3	1	3	19	4.8
	4	LP	4	4	4	4	3	2	2	12.6	7.4
4	1	LP	4	3	3	4	2	0	2	8	26.6
	2										
	3	LP	4	2	0	4	1	0	3	28	11.2
5	1	LP	4	N/A*	N/A*	4	N/A*	0	3	0	30
	2										
	3	LP	4	4	4	3	3	3	3	5	13
	4	LP	4	4	4	2	3	2	3	7	10.7
6	1	LP	4	4	4	1	1	2	2	10	12.6
	2										
	3	LP	1	0	0	0	0	2	0	2	5.6
7	1	LP	4	3	3	2	1	3	2	15	10.3
	2										
	3	LP	4	3	4	1	3	1	3	9	6.5
	4	LP	4	4	4	2	4	2	2	2.4	2
8	1	LP	4	2	3	3	2	2	2	3	2.2
	2										
	3	LP	4	2	0	0	0	1	2	2	6.6
	4	LP	3	3	3	0	2	1	2	7	7

All cats had supernatant treatment and biopsies on visits 1 (V1) and V3 (approximately on days 1 and 90), and four cats had an additional treatment and biopsy on V4 (approximately on day 180).

LP, lymphocytic-plasmacytic.

^ Grading scheme: (0) absent; (1) minimal—scant perivascular inflammatory infiltrate or <10% stroma/epithelium affected; (2) mild—10–25% of the stroma/epithelium affected; (3) moderate—25–50% of the stroma/epithelium affected; (4) marked/severe— > 50% of the stroma/epithelium affected.

# Grading scheme: (0) absent; (1) few/minimal; (2) mildly increased; (3) moderately increased; (4) markedly/severely increased.

*N/A: No intact epithelium in sections examined for assessment for exocytosis, erosion, or epithelial hyperplasia.

+ phf: Number of cells per 0.237mm^2^.

Severity of inflammation, erosion, ulceration, epithelial hyperplasia, stromal oedema, granulation tissue, or fibrosis were each graded from 0 to 4 using a semiquantitative system: absent (0), minimal—scant perivascular inflammatory infiltrate or <10% stroma/epithelium affected (1), mild—10–25% of the stroma/epithelium affected (2), moderate—25–50% of the stroma/epithelium affected (3), and marked/severe— > 50% of the stroma/epithelium affected (4). Cell types (eosinophils, neutrophils, globule leukocytes, small lymphocytes, and plasma cells) were each graded from 0 to 4 using a semiquantitative system: absent (0), few/minimal (1), mildly increased (2), moderately increased (3), or markedly/severely increased (4) (Table 1).

Assessment and quantification of mast cells was performed on TB-stained slides. The number of positively stained cells (i.e., cells containing purple cytoplasmic granules) within the epithelium and lamina propria was determined from 10 randomly selected microscopic high-power fields (hpf) at x 400 magnification (0.237mm^2^ field of view), or the maximum number of microscopic high-power fields present. The average number of positively stained cells was then expressed per hpf or 0.237 mm^2^ of tissue. Assessment and quantification of Mott cells was performed on H&E-stained slides similar to mast cells described above (Table 1).

#### 2.4.2 Statistical analysis

Changes in SDAI and weight for all eight cats up to V4 were compared using the Wilcoxon signed-rank test.

## 3 Results

### 3.1 Study population

Out of eight cats reported in the study, four had undergone partial mouth extractions and four had undergone total mouth extractions, all over 6 months prior to the treatment. Cats were of various ages ranging from 2 to 13 years old, four cats were below 4 years old, and four cats were above 8 years old. Most cats were domestic short hair. There were five females and three males, and all were desexed. Three cats tested positive for FIV, and none tested positive for FeLV. The data for the study population are shown in Table 2.

**Table 2:** Study population, signalment, and clinicopathological data from cats suffering from FCGS treated with MSC secretome.

Cat ID	Visit number	Age (years)	Sex	FIV	Weight (kg)	SDAI	Dental extractions	GLOB (g/dL)	LEUK (k/μl)	NEU (k/μl)	LYM (k/μl)
1	1	2.2	Female	+	2.5	18	Full	83	5.7	3.4	1.8
	2				2.7	15		60	6.5	4.5	1.8
	3				2.8	15		61	7.8	4.6	2.7
2	1	2.4	Female	+	3.1	22	Full	62	7.6	4.1	2.6
	2				3.2	21		61	7.4	4.4	2.2
	3				3.2	16		N/A	N/A	N/A	N/A
	4				3.2	15		58	12	10	0.9
3	1	9.7	Male	−	6.4	25	Full	63	10.9	6.4	3.1
	2				6.8	25		48	17	15	1.1
	3				6.4	19		52	15	12	2.7
	4				5.7	20		53	16	12	1.9
4	1	11	Male	−	5.1	27	Full	51	16	14	1.1
	2				5.2	25		44	18.8	16	1.1
	3				4.6	22		46	30	26	1.2
5	1	2.9	Female	−	2.9	25	Partial	63	10.4	7	2.7
	2				2.8	24		58	12	8.5	2.8
	3				2.7	25		70	8.6	6.1	1.8
	4				2.7	20		64	7.2	4	2.7
6	1	3.8	Male	−	5.4	21	Partial	57	10.7	6.2	3.5
	2				5.6	14		53	11	5.8	3.9
	3				6	1		41	6.4	4.4	3.9
7	1	8	Female	+	4	25	Partial	62	14	11	1.9
	2				4.3	20		63	12	9.7	2.1
	3				4.4	15		67	14	13	0.7
	4				4.5	15		73	15	12	1.8
8	1	13	Female	−	3.4	18	Partial	54	19	12	0.4
	2				3.3	16		49	9.9	5.3	1.5
	3				3.4	4		54	13	7	3.6
	4				3.5	6		41	11.4	7.6	1.4

All cats were domestic short hair (DSH) except for cats 6 and 8 that were British short hair (BSH) and domestic long hair (DLH), respectively. All cats had supernatant treatments on visits 1 (V1), V3, and V4 (approximately on days 1, 90, and 180).

### 3.2 SDAI and weight

The SDAI improved in all eight cats with drops in the score of 5 to 20 points. The highest score at V1 was 28, and the lowest was 18. Following treatment with MSC secretome, the highest SDAI was 22 and the lowest was 1 (Table 2).

The weight of the cats was monitored in every visit and increased in 62.5% (5/8) of the cats. The remaining 37.5% (3/8) of cats decreased in weight despite improvement in SDAI (Table 2).

### 3.3 Blood test results

There was a decrease in serum GLOB in 75% (6/8) of cats when comparing V1 to V3 or V4 if the cat had four visits. The highest decreases were 14 units, followed by 8 units, and occurred in the two cats that showed resolution of clinical signs. Two cats had an increase in globulins. Weight increase and globulin decrease occurred in 62% (5/8) of the cats. Leukocyte count decreased in 50% (4/8) of the cats and increased in 50% (4/8) of the cats with fluctuations throughout the visits. Neutrophils increased in 62.5% (5/8) of the cats and decreased in the remaining 37.5% (3/8) with decreases in the two cats that had clinical resolution of signs. Lymphocytes, however, increased in the two cats that showed resolution of clinical signs and in one cat that did not improve clinically and lost weight but decreased in the remaining 62.5% (5/8) of cats. The results are shown in Table 2.

### 3.4 Histopathology

**Figure 2:**
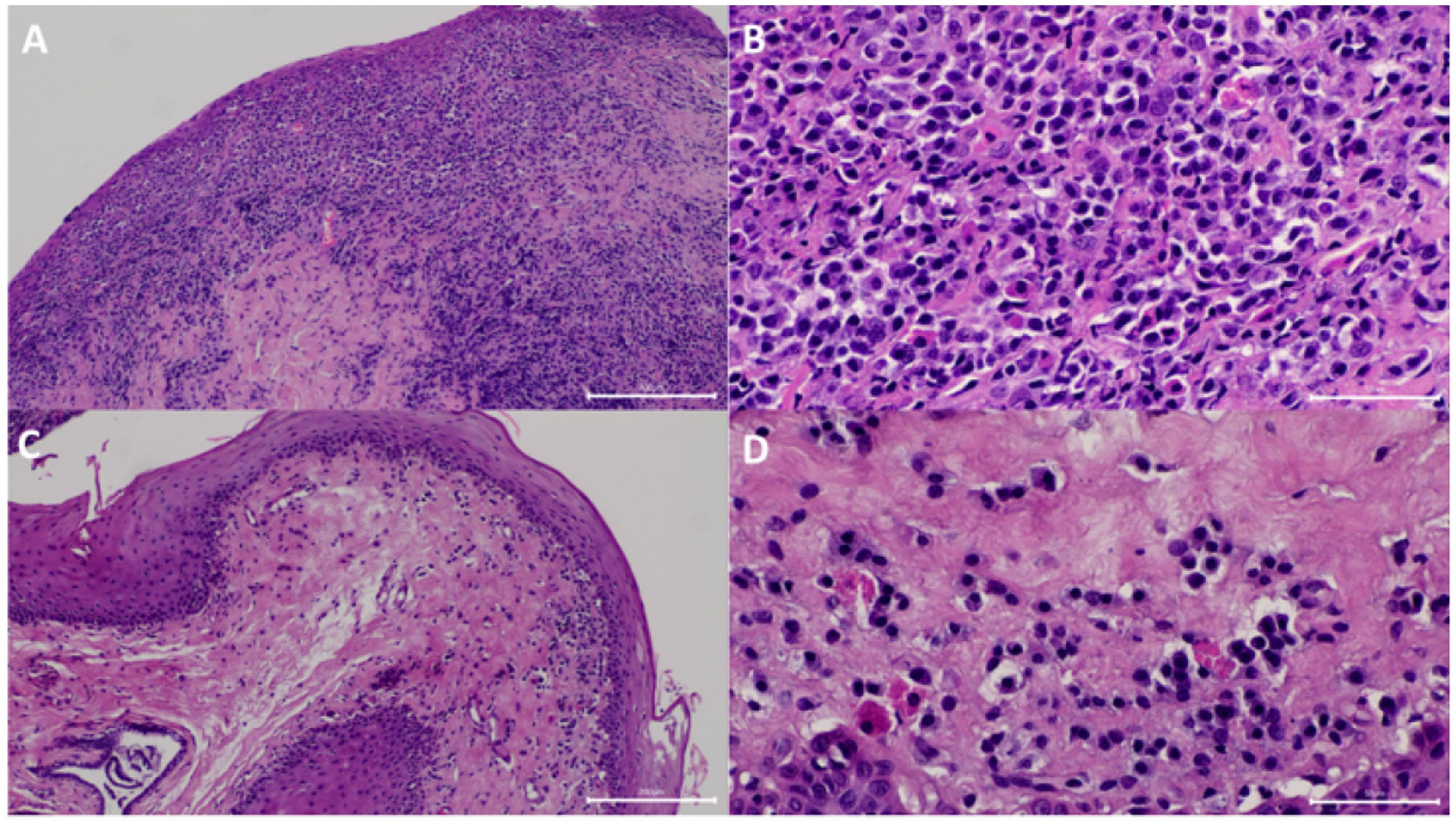
Samples from one of the cats that showed resolution of clinical signs. **(A)** At visit 1 (V1), the submucosa has marked diffuse inflammatory infiltrate. The mucosa was eroded. HE 100x. **(B)** Higher magnification of panel **(A)** showing the infiltrate was predominantly composed of plasma cells, fewer lymphocytes, a few neutrophils, and Mott cells HE 400x. **(C)** At V3, the submucosal inflammatory infiltrate was significantly decreased. HE 100x. **(D)** Higher magnification of panel **(C)** showing the decreased inflammatory infiltrate. HE 400x.

**Figure 3:**
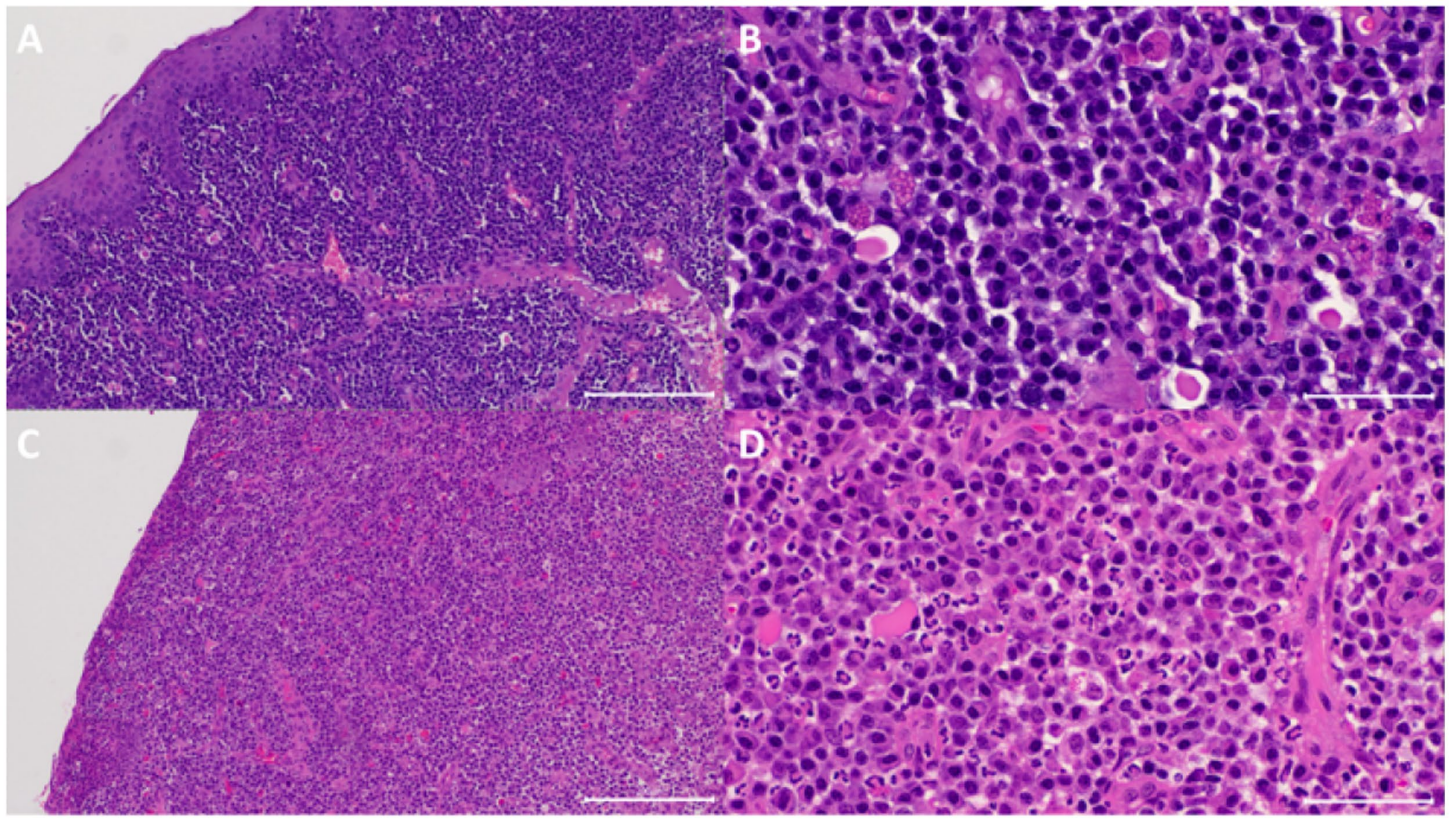
Sample from one of the cats that did not show clinical resolution of clinical signs. **(A)** At visit 1 (V1), the submucosa has marked diffuse inflammatory infiltrate. The mucosa was eroded. HE 100x. **(B)** Higher magnification of panel **(A)** showing the infiltrate was predominantly composed of plasma cells, fewer lymphocytes, a few neutrophils and Mott cells. HE 400x. **(C)** At V3, the submucosal inflammatory infiltrate was similar in terms of composition and density. HE 100x. **(D)** Higher magnification of panel **(C)** showing the similar dense inflammatory infiltrate. HE 400x.

All analysed samples had lymphocytic-plasmacytic infiltration as the histopathological principal change. The severity of infiltration was marked (grade 4+) on V1 for all the cases and changed to minimal (grade 1+) or mild to moderate (grade 3+), respectively, in the two cats that showed resolution of clinical signs on V3 (Figure 2) but remained as marked (grade 4+) in the rest of the cats (Figure 3).

In the two cats that showed resolution of clinical signs, there was positive correlation between histopathological changes and clinical signs in terms of improvement in severity of inflammation (from both marked grade 4 + on V1 to minimal grade 1 + or mild to moderate grade 3 + on V3, respectively), ulceration (from both grade 1 + and 3 + on V1 to absent grade 0 both on V3, respectively), epithelial hyperplasia (from grade 1 and grade 2 on V1 to absent grade 0 both on V3, respectively), and number of Mott cells (both had decreased Mott cell on V3) (Table 1).

On V3, Mott cells were decreased in 75% (6/8) of the cats, while 87.5% of (7/8) cats had decreased mast cell infiltrate. Increased severity of neutrophilic infiltrate was observed in 50% (4/8) of the cats on V3. One cat had similar degree of neutrophilic infiltrate, compared to V1 (Table 1).

Other histopathological changes observed included absence of globular leukocytes from all samples, one sample contained rare eosinophils in the representative section examined, presence of exocytosis composed of lymphocytes and neutrophils when intact epithelium was present, ulcerations, and erosions together with ulcerations (Table 1). Epithelial hyperplasia was also appreciated on presentation and became inapparent in the two cats that showed clinical resolution of signs. Stromal oedema was often present and appeared unrelated to severity of inflammation. Granulation tissue or fibrosis was not appreciated in all samples examined.

### 3.5 Side effects

No cat had any adverse effect directly associated with the use of MSC secretome. Two cats had hypoglycaemia from prolonged fasting above 15 h. Slow recovery after atipamezole reversal occurred in one of the cats that had hypoglycaemia and hypotension and was kept in hospital overnight for fluid therapy and dextrose supplementation.

### 3.6 Statistical analysis

**Figure 4:**
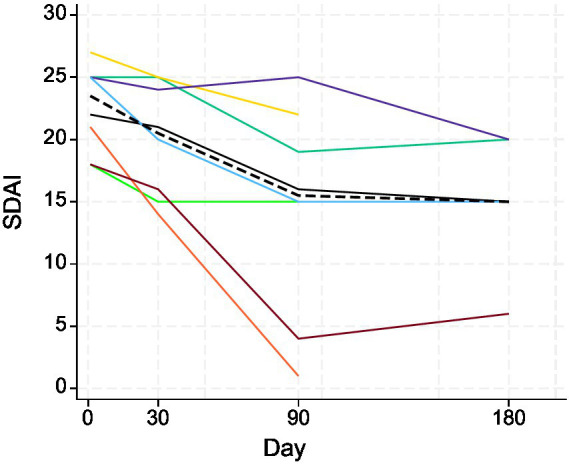
Changes in SDAIs of the eight study cats (different colors) following treatment with MSC secretome. The dashed black line represents the median. Days 1, 30, 90, and 180 correspond to V1, V2, V3, and V4. Visit 4 measures were only available for five cats.

**Figure 5:**
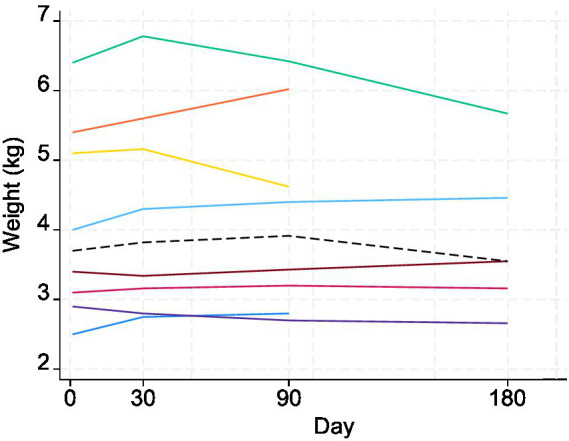
Changes in weight (kg) of the eight study cats (different colors) following treatment with MSC secretome. The dashed black line represents the median. Days 1, 30, 90, and 180 correspond to V1, V2, V3, and V4. Visit 4 measures were only available for five cats.

The SDAI showed a decreasing trend following treatment up to V3 around day 90 (Figure 4). In the Wilcoxon signed-rank tests, differences in SDAI between V1, V2, and V3 were all statistically significant: V1 and V2 (*p* = 0.0138); V2 and V3 (*p* = 0.0294); V1 and V3 (*p* = 0.0139). The weight of cats did not show any clear trend during the study period (Figure 5). There was no statistically significant difference in the weight of cats (up to V3) between the days of measurement (all *p*-values > 0.05).

## 4 Discussion

The treatment of MSCs for FCGS has become an attractive alternative among clinicians for this insidious disease when no other alternatives are effective or available. The availability of MSC secretome in Hong Kong prompted curiosity among clinicians regarding its clinical benefits. This study is a case series that describes the treatment of eight cats with MSC secretome for the treatment of FCGS following partial or full mouth dental extractions.

Although there is abundant literature supporting the use of MSCs for FCGS (9, 11, 13, 14), to the authors’ knowledge, xenogeneic MSC secretome has not been used in cats before. Cell-free secretome is likely to exert similar paracrine effects but reduce potential complications and side effects. The effect of secretome-factors on monocytes, T cells, B cells, TGF beta, PGE2, IL-10, and TSG-6 among others would have been beneficial, leading to angiogenesis, wound healing, inhibiting proliferation, and modulating inflammation (15). The lack of cells has the advantage of avoiding potential side effects recognized with the use of allogeneic transplants such as pulmonary embolisms and limb oedema (11, 36). These complications occur due to formation of alloantibodies in B cells consequent to major histocompatibility complexes (MHC) I and II leading to cytotoxicity or humoral responses (37). In addition to reducing potential side effects, the secretome is easily preserved by freezing it without the need for cryopreservatives and is convenient for its potential immediate availability. We therefore highlight in this report the potential advantages using this novel therapy in cats with FCGS.

Retroviral infection status was available for all cats, three of which were reported FIV-positive. Retroviral status has not been proven to be directly associated with FCGS though (38). Although none of the FIV-positive cats showed significant clinical improvement after treatment, all three cats gained weight and two of them had a decrease in GLOB. Immune depression and infections are a consequence of FIV in cats, and although association of bacteria with FCGS might be supported (39), a multifactorial aetiology remains in favour (8, 38, 40). Feline calicivirus (FCV) was not reported, but the authors acknowledge that infection might be present in most cats affected by FCGS, as this has been reported in up to 90% in other studies (41).

The SDAI improved in all treated patients. This grade score is based on a questionnaire and an oral exam. On all occasions, the exam was done or repeated after sedation often with medetomidine. The use of alfa-2-agonists might reduce hyperaemia of the affected oral mucosa due to vasoconstriction, but the proliferative lesions would continue to be visible even if considerably less congested. This would have still limited the accuracy of the SDAI score. The retrospective nature of this study limits the standardization of sedation protocols that might have changed the appearance of oral mucosa; similarly, we cannot rule out interobserver discrepancies in retrospective studies such as this; however, the clinicians conducting treatments used the same protocols. The owners’ perception is included in these questionnaires, and improvements were always reported even if the oral visual exam showed no significant clinical improvement of the lesions. Often the clients reported an improvement shortly after the injection. This might be supported by greater improvements shown from V1 to V2 compared to V3 or V4 (Figures 4, 5). The decrease in weight of 37.5% (3/8) of the cats despite improvement of SDAI highlights the challenges on interpreting both questionnaires and oral exam though.

Since the cases were all cats treated at VMC, all blood tests were also analysed at VMC in house or at its affiliated laboratory, VDL. Globulin values were considered important as they are indicative of chronic inflammatory processes and have been associated with FCGS (13, 42). The highest decrease of GLOB occurred in one of the cats that had resolution of clinical signs; however, other cats including cats that had lost weight or had more moderate decrease of SDAI had also decreased GLOB. Despite lack of clinical correlation, numbers of Mott cells were decreased in 75% (6/8) of the cats on V3, which correlated with the decrease of serum globulin in 75% of cats (6/8). Mott cells are plasma cells with immunoglobulins packed in the cytoplasm and are often noted in inflammatory lesions involving reactive plasmacytosis (43). MSC secretome may have an effect on Mott cell formation. The decrease of Mott cells in the lesions may contribute partly to the decrease of serum globulin observed in this study. The role of Mott cells in the pathogenesis of FCGS remains uncertain. Decreased GLOB and increased weight were reported in 50% (4/8) of cats suggesting a positive immunomodulatory effect downregulating proinflammatory factors leading to lower globulinaemia, potentially improving appetite and weight gain in a subsection of cats. Higher SDAI scores of 20 or above were observed only in cats that lost weight suggesting a positive correlation between weight loss and increased SDAI. The different stages of disease in each cat were an important limitation as cats with less severe disease might have responded better to the treatment.

Leukocyte, neutrophil, and lymphocyte counts fluctuated throughout the visits. Similarly, on V3, 50% (4/8) of the cats had increased severity of neutrophilic infiltrate compared to V1 which appears to be unrelated to the degree or severity of ulcerations or erosions on histology. Rapid short-term changes may occur daily in response to daily insults; in addition, interpretation of these fluctuations is challenging and is poorly specific for affected body systems. We therefore suggest their measurement would be poorly correlated with treatment response, especially when measured months apart. Daily measurements following treatments might give a better indication of whether an anti-inflammatory response has occurred.

All biopsies were reviewed by the same pathologists decreasing interpretation variabilities. Histological changes were similar to those described in previously published literature (44). In the two cats with resolution of clinical signs, there was positive correlation between histopathological changes and clinical signs with reduction in severity of inflammation, ulceration, epithelial hyperplasia, and number of Mott cells. For the rest of the cats with milder clinical improvement, it was not unexpected that the histology changes remained similar after treatment.

Previous studies have reported an increase in the number of mast cells in the gingiva of cats affected with feline chronic gingivostomatitis (45), which is consistent with our findings where 87.5% (7/8) of the cats had increased mast cells on V1; in addition, the same 87.5% of (7/8) of cats had decreased mast cell infiltrate on V3 in our study. MSC secretome may pose an effect on mast cell infiltrate. However, despite the decrease in mast cells, the severity of the inflammation remained similar in most cats and there was no apparent correlation with the cats that showed resolution of clinical signs. Mast cells appear less likely to have a major role on the pathogenesis of FCGS.

Some limitations of the study included aspects of its design. The small number of cases in this study was collected from one hospital; therefore, the changes observed might not be representative for a wider population of cats or cats seen in different medical settings. Larger cohorts in prospective blinded control studies are suggested for increasing statistical power, and this would have been ideal to support the significance of the results observed in this study, but the lack of eligible candidates and the reticence of clients to participate in studies as controls precluded this stronger design. To overcome some of the design limitations, only cases that had met a more standardized treatment and monitoring were included, and a before-after design study could have also be explored in this particular condition. Due to the progressive nature of FCGS, the authors consider the low number of patients might be still significant, as no spontaneous recovery has ever been reported. Therefore, this report might still showcase a positive effect of a novel therapeutic approach to an incurable and debilitating disease.

## 5 Conclusion

This is the first report of xenogeneic MSC secretome used for the treatment of FCGS. The administration of secretome appeared to be well tolerated and had not known directly associated side effects. Although the clinical lesions did not improve in most (75%) cats, there was improvement in analytes such as GLOB (75%), as well as weight (62.5%) with confirmatory histopathological favorable changes in 25% of the cats. The use of MSC secretome might be beneficial as an additional or alternative treatment for a subsector of cats suffering from FCGS; however, its administration route and dose needs to be better studied. The limited collected evidence and the small sample size included in this study warrants the need for further studies with larger number of cats and prospective control groups before we can establish recommendations for its use in a clinical setting.

## Data Availability

The raw data supporting the conclusions of this article will be made available by the authors, without undue reservation.
